# Variations in acrylamide content of homemade biscuits with the addition of chia (ground seeds and flour)^☆^

**DOI:** 10.1016/j.fochx.2025.103172

**Published:** 2025-10-15

**Authors:** Hind El Hadri, Ivana Blažević, Ivana Bianchi, Otmar Geiss, Josefa Barrero-Moreno

**Affiliations:** European Commission, Joint Research Centre (JRC), Ispra, Italy

**Keywords:** Chia seeds, Acrylamide, Homemade biscuits, Baking, Novel food, Low-moisture

## Abstract

Homemade food constitutes an important component of people's diets, yet remains understudied for heat-processed contaminants. This study replicated realistic home-baking conditions to evaluate acrylamide variability. Ground chia seeds or defatted chia flour (10 %) were incorporated into biscuits and compared to 100 % wheat-flour controls. Three baking sessions were conducted with nine independent bakers producing 24 batches. Each session involved three to five bakers using the same recipe while varying parameters such as oven mode, ingredient type, and biscuit shape. Acrylamide concentrations ranged from 2.4 to 37 μg kg^−1^, below the EU benchmark level of 350 μg kg^−1^. Adding 10 % chia did not impact acrylamide levels. Key influencing parameters included moisture content, pH, and physical parameters (thickness, mass, and shape). Water content in both biscuits and oven atmosphere critically impacted acrylamide formation. This suggests that careful control of baking moisture could be a practical mitigation strategy at household level.

## Highlights

• Comparing acrylamide content in homemade biscuits made under real-life scenarios.
• Acrylamide in chia biscuits prepared by nine bakers applying various conditions.
• Moisture identified as a key factor influencing acrylamide formation.
• 10 % chia seed flour shown not to increase significantly acrylamide formation risk.

## 1 Introduction

Acrylamide (AA) is a chemical that forms naturally in some foods when they are heat-treated (fried, baked or roasted) at high temperature. Research has shown that reducing sugars and specific amino acids, particularly asparagine, contribute to the formation of acrylamide in foods through the Maillard reaction (Mottram et al., 2002; Stadler & Gökmen, 2024; Zyzak et al., 2003). AA has been identified as possibly carcinogenic and neurotoxic (EFSA, 2015; IARC, 1994). The main sources of AA exposure in food are potato-based products, cereal-based products, and coffee (Claus et al., 2008; Kocadağlı & Gökmen, 2022; Krishnakumar & Visvanathan, 2014; Medeiros Vinci et al., 2012). Timmermann et al. (2021) reviewed dietary AA intake data from 68 original studies across 26 countries and found that median AA intakes ranged from 0.02 to 1.53 μg kg^−1^ body weight/day. Their analysis showed that estimated AA intakes of children were up to three times higher than those of adults, probably due to their higher consumption of starchy foods relative to body weight (Timmermann et al., 2021). Several epidemiological studies suggest an increased risk for certain gynaecological cancers (Bellicha et al., 2022) and adverse effects of AA exposure on foetal growth (Hogervorst et al., 2022). A study by Pekmezci and Basaran (2023) on dietary heat-treatment contaminants exposure found that French fries, known for having high levels of acrylamide, were associated with a higher risk score for cancers in both the urinary and reproductive systems compared to other cancer types (Pekmezci & Basaran, 2023). Despite these findings, the current evidence on the relationship between dietary AA exposure and cancer risk remains limited (Başaran et al., 2023; Filippini et al., 2022). Therefore, it is crucial to be aware of the potential health risks associated with AA consumption. Mitigation strategies can be implemented to reduce AA formation. Adjusting factors such as temperature, cooking time, water content, pH levels, and the use of acrylamide-reducing agents like asparaginase can be effective. Additionally, using raw materials with lower sugar or asparagine content can help minimise AA formation (EU Regulation 2017/2158, 2017; FDA, 2016; FoodDrinkEurope, 2019).

Several studies showed a decrease in AA occurrence in several food products since AA discovery in food in 2002 (Abt et al., 2019; Mesías et al., 2019; Powers et al., 2021). While industrial food producers may attempt to minimise AA formation in their products, home cooking is more subject to variability and to higher AA levels, as consumers do not follow a standardised procedure to reduce AA. Moreover, they tend to overcook their food as shown by a study by Mesías et al. (2020), which assessed the impact of consumer cooking practices on AA formation during the preparation of French fries in Spanish households. Notably, the study found a discrepancy between participants' perceptions and actual cooking reality, with 78 % of participants classifying their fried potatoes as “golden” whereas a harmonised classification indicated that nearly 40 % of these samples were, in fact, overcooked (Mesías et al., 2020). It is considered that half of the AA exposure results from cooking in private homes or in restaurants in contrast to industrially processed foods (Grob, 2007). For example, Shakeri et al. (2019) found that AA formation was nearly twice as high in the domestic preparations of traditional Iranian cookies (Kolompeh) as in the industrial processes (Shakeri et al., 2019), highlighting the significant contribution of home cooking to AA exposure.

Incorporating new ingredients into traditional European recipes could potentially change the amount of AA formed during food processing. Chia seeds (*Salvia hispanica L*.) are a nutrient-rich ingredient, approved as a novel food ingredient in various categories, both with and without heating processes. They can be used in non-heated items like nut mixes, confectionery, and beverages, as well as in heated products such as bread, baked goods, breakfast cereals, and sterilised ready-to-eat meals, with specific maximum usage levels (maximum 10 %) (EU Regulation 2017/2470, 2017). Additionally, partially defatted chia seed powders, which are high in protein and fibre, are approved for use in non-heated foodstuffs like juice, food supplements, and confectionery and heated foodstuffs such as cakes, bread and pasta-based products (EU Implementing Regulation 2020/500, 2020; EU Implementing Regulation 2023/2214, 2023), however, not yet in low-humidity foodstuffs (e.g. crackers, biscuits). The impact of chia seed addition on AA levels in food products was assessed in bread (Bartkiene et al., 2023; EFSA Panel on Nutrition et al., 2023; Galluzzo et al., 2021) and biscuits (Hölzle et al., 2025; Mesías et al., 2016; Mesías et al., 2023). In bread, the addition of 10 to 30 % chia seeds showed a significant reduction in AA formation (17 to 34 % less than the control) in the study by Bartkiene et al. (2023), while Galluzzo et al. (2021) found no significant differences in AA levels between control samples and breads with chia seeds addition (2 to 10 %). The lower AA amounts in chia-enriched breads may be attributed to a higher water retention of chia (EFSA Panel on Nutrition et al., 2023), lower levels of reducing sugars and the presence of phenolic compounds in chia seeds (Bartkiene et al., 2023; Galluzzo et al., 2021; Liu et al., 2015). In biscuits, the addition of chia seeds at levels up to 20 % does not significantly increase the AA content (Hölzle et al., 2025). However, adding ground chia or defatted ground chia (ranging from 5 to 20 %) often resulted in higher amounts of AA compared to the control (up to 8 times) (Hölzle et al., 2025; Mesías et al., 2016; Mesías et al., 2023).

The potential for AA formation is highest in low-moisture products such as biscuits and crackers. The amount of water in the baked goods affects the food temperature, with the evaporating water allowing the temperature to not exceed 100 °C, resulting in negligible AA formation, particularly in the inner part of the biscuit (Adimas et al., 2024). However, more favourable conditions for AA production are created on the outer part of the food, where the temperature is higher and moisture is lower (Açar & Gökmen, 2009; Pietropaoli et al., 2022; Schouten et al., 2022).

Home baking is inherently subject to variation, with factors such as oven differences, ingredient weighing errors, dough thickness inconsistencies, and subjective liquid additions to achieve the right dough consistency; all of which contribute to unpredictability. With chia seeds are becoming increasingly popular in home cooking, this study aims to investigate the impact of partial wheat flour replacement on AA formation in homemade biscuits. A series of home baking tests were carried out using the same recipe with multiple bakers and various kitchen setups to prepare control biscuits (without chia) and biscuits with 10 % chia substitution.

## 2 Materials and methods

### 2.1 Materials

The ingredients (flour, sunflower oil, sugar, baking powder, salt and water) were bought from a local Italian supermarket. The flour is type 00, which is the most finely ground wheat flour that is commonly used in Italian cuisine for sweets. It may absorb less water than other type of flour (coarser) (Cristiano et al., 2019). The water used for cooking came from mineral water bottles (San Benedetto). The sugar was a supermarket's own brand product made with beet, and as such was primarily composed of sucrose. The baking powder used was composed of disodium diphosphate (E 450), sodium carbonate (E 500), starch, sodium, potassium, and calcium salts of fatty acids (E 470a), and flavours. Two chia products were used: chia seeds and defatted chia flour. The main differences were in protein (18 g vs. 30 g), carbohydrates (0.8 g vs. 9 g) and fat content (36 g vs. 8 g) (Table S1).

Ultrapure water (18 MΩ.cm) was supplied by a Millipore water purification system (Merck, Darmstadt, Germany) and was used for all sample preparations and LC mobile phase. Formic acid and LC-MS grade (≥99.9 %) methanol for mobile phase preparation were purchased from Sigma-Aldrich (Milan, Italy). Acrylamide (AA) powder (>99 %) and deuterated (D3) acrylamide (d3-AA) (>99 %) standard solution, both purchased from Sigma-Aldrich, were used to prepare calibration standards and the internal standard (ISTD), respectively.

### 2.2 Formulation

Formulation of the biscuits was adapted from the recipes found in the literature (Duarte et al., 2024; Goswami & Awasthi, 2022; Hegazy, 2019; Joshi & Awasthi, 2020; Mesías et al., 2016) and online (see some websites in SI). The objective was to use a simple formulation with minimal ingredients, excluding milk and shortening. Table 1 shows the ingredients and quantities used for making biscuits at home. The dough preparation process started by combining the dry ingredients, comprising flour, sugar, baking powder, and salt with oil in a bowl. The ingredients were initially mixed using a spoon, followed by manual manipulation to form a rough dough. Water was then gradually added to the mixture, and the dough was worked until it reached a supple, homogeneous consistency. Care was taken to avoid over-working the dough, which could lead to undesirable texture and structure. The dough was subsequently shaped into a ball, wrapped in film, and refrigerated for 15 min to allow relaxation.

**Table 1:** Ingredient list and corresponding amounts.

Ingredients	Biscuit
Flour (wheat and chia)	Control (**CTRL**): 100 g wheat Test-sample (**10 %-Chia**): 90 g wheat +10 g ground chia seeds or defatted chia flour
Sugar	35 g
Fat (sunflower oil)	25 g
Baking powder	1 g
Salt	1.2 g
Water	20–35 g as required for the dough consistency Amount varied depending on the baker

The chilled dough was then rolled out to a thickness of 3–6 mm with a rolling pin and cut into round shapes using 5 cm cookie cutters. The oven was preheated to 180 °C either in static mode or in convection mode, using both top and bottom heating elements. The cut biscuits were placed on a piece of baking paper positioned on an oven baking tray, which was then inserted into the middle of the oven. The biscuits were baked for 12 min. After baking, the biscuits were removed from the oven and left on the tray to cool until they could be handled. They were then transferred to a wire rack to undergo complete cooling. Once cooled, the biscuits were stored in a sealed plastic bag until further processing and analysis.

Three sets of baking experiments were conducted by multiple bakers and are summarised in Table 2. Specifically, the original biscuit recipe and ingredients (described in section 2.1) remained unchanged between the first and second set of experiments. However, two changes were made: the oven mode changed from static to convection and the precision in measuring thickness and the masses of baking powder and salt was improved during the preparation phase. In Set #3, bakers were provided with the same recipe to obtain the same biscuit composition (i.e. flour, oil, sugar, etc.); however, they had the freedom to prepare the biscuits according to their usual practices, i.e., selecting their own ingredients (Table S2) baking as long as they deemed necessary, based on their own judgment.

**Table 2:** Experimental design variations across biscuit baking sets.

Parameter	Set #1	Set #2	Set #3
Ingredients used among bakers	Same ingredients (section 2.1)	Same ingredients (section 2.1)	Different ingredients (Table S2)
Chia type	Ground chia seed	Ground chia seed	Defatted chia flour
Oven mode	Static	Convection	Convection or static
Baking time	12 min	12 min	12–18 min according to the baker judgment
Thickness	3–6 mm – Rolling pin without spacers	5 mm – Rolling pin with adjustable thickness spacers	Around 5 mm Rolling pin without spacers
Shape	Round	Round	Variable (round, star, scalloped circle)
Weight precision	1 g for all ingredients	0.01 g for baking powder and salt 1 g for the rest of ingredients	1 g for all ingredients
Number of bakers	3 (baker A, B and C)	5 (baker A, B, C, D and E)	4 (baker F, G, H and I)
Number of batches per set	6 batches (3 CTRL, 3 10 %-Chia)	10 batches (5 CTRL, 5 10 %-Chia)	8 batches (4 CTRL, 4 10 %-Chia)

### 2.3 Physico-chemical analysis

#### 2.3.1 Physical parameters

For each baking set, several physical parameters were measured:

- Mass of each baked biscuit was determined using a digital balance at 0.01 g precision (Mettler Toledo PB3002-S, Milan, Italy) and then averaged.
- Diameter of biscuits was determined by placing six biscuits edge to edge and measuring the total length using a measuring scale. The biscuits were rotated at an angle of 90° and the diameter was measured again. Two sets of measurements were performed, resulting in four measurements for each run, which were then averaged and divided by the number of biscuits (values are presented as mean ± S·D).
- Thickness of biscuits was determined by stacking six biscuits on top of each other, measuring the height using a Vernier calliper and dividing by six. The measurement was performed on four sets of biscuits from the same batch and values are presented as mean ± S.D.
- The spread ratio was calculated by dividing the average diameter by the average thickness.
- Percentage of weight loss (WL%) during cooking was determined by measuring the mass of dough before baking and the mass of biscuits after baking and calculated as $\mathrm{WL}\%=100-\left(\frac{{\mathrm{mass}}_{\mathrm{after baking}}}{{\mathrm{mass}}_{\mathrm{before baking}}}\times 100\right)$

#### 2.3.2 Moisture determination

Moisture was determined gravimetrically by weighing the food sample before and after heating in a convection oven (ARGOLAB TCF 200, Carpi, Italy) at 105 °C for 24 h (Mesías et al., 2023). The moisture content (MC) was determined as $\mathrm{MC}\%=\frac{{\mathrm{mass}}_{\mathrm{Initial}}-{\mathrm{mass}}_{\mathrm{Dried}}}{{\mathrm{mass}}_{\mathrm{Initial}}}\times 100$ where mass_initial_ is the mass of the food sample before heating and mass_dried_ is the mass of the food sample after heating. The difference represents the mass of the water initially present in the biscuits.

#### 2.3.3 pH determination

The supernatant from the third step of the SPE extraction method (section 2.3.4 and Fig. S1) was used to measure pH. The pH was determined using a pH 80 pHmeter (XS instruments, Carpi, Italy).

#### 2.3.4 Acrylamide determination

To ensure a representative sample and account for variations in cooking that may occur due to differences in oven placement, four to six of the baked biscuits from each batch were selected and ground using a grinder: IKA A11 basic (Staufen, Germany). The extraction method used for AA is based on the EN 16618:2015 standard method and is described in Fig. S1. Briefly, 40 mL of water was added to 2 g of ground food sample and d3-AA ISTD was added to achieve a final concentration of 10 μg L^−1^. To homogenise the samples, the mixture was first shaken by hand, then vortexed for 10–15 s (VELP Scientifica, Usmate Velate, Italy), followed by mechanical shaking for 60 min (rotator SB3, Stuart). The samples were then centrifuged at 3600 *g* for 20 min at 10 °C using an Eppendorf 5920R centrifuge (Milan, Italy). 10 mL of the supernatant was taken for the solid phase extraction (SPE). Two SPE columns were used to clean-up and concentrate AA from food (Column 1: ISOLUTE® Multimode 1 g/6 mL, column 2: ISOLUTE® ENV+ 500 mg/6 mL) (Biotage, Uppsala, Sweden). SPE processing was performed using a positive pressure manifold (UCT, Bristol, USA). The final eluate (approximately 2 mL) was evaporated using a sample concentrator with a nitrogen flow (Techne dri-block DB-3D) reducing the volume to around 0.5 mL. This concentrate was then transferred to a liquid chromatography vial. The final ISTD concentration varied with the volume, and was, on average, 130 μg kg^−1^.

The extracted samples were analysed using liquid chromatography-tandem mass spectrometry (LC-MS/MS) with a 1290 infinity II LC system (Agilent, Cernusco sul Naviglio, Italy) and an Ultivo Triple Quadrupole mass spectrometer (Agilent). The LC conditions were adapted from the EN 16618:2015 standard method while the MS conditions were optimised. The LC-MS/MS parameters are described in Table S3. Analytical separation was performed using a porous graphitic carbon column (Hypercarb, Thermo Fisher Scientific, Rodano, Italy). Mobile phase A was 0.1 % formic acid in water and mobile phase B was 100 % methanol at a flow rate of 0.3 mL min^−1^. The retention time of AA occurred within the initial isocratic elution phase of the gradient (< 4 min). The LC eluent was directed to the MS interface within the retention time window 1–6 min using a diverter to prevent contamination of the MS. The total run time of the chromatograms was 10 min. Deuterated acrylamide (d3-AA) was used as internal standard at a concentration of ca. 40 μg kg^−1^ in the calibration samples. The AA standards were prepared in water, with AA concentrations for the calibration ranging from 0.5 μg L^−1^ to 50 μg L^−1^. The coefficient of determination (R^2^) ranged from 0.9998 to 1.0000. The limit of detection (LOD) and limit of quantification (LOQ) of the method were determined by analysing a blank sample multiple times using the complete sample preparation process. The LOD and LOQ values were found to be 0.06 μg kg^−1^ and 0.12 μg kg^−1^, respectively. Each sample was extracted at least twice, and the uncertainty was calculated as the standard deviation of these replicates.

### 2.4 Statistical method

Statistical analysis was performed using DataTab software (https://datatab.net). The significance of differences between groups was determined using independent two-sample *t*-test. Pearson correlation coefficients were calculated to assess the relationships between variables. A *p*-value of <0.05 was considered statistically significant.

## 3 Results and discussion

### 3.1 Biscuit physical properties

The physical properties of the biscuits were determined for each baking set, with distinct differences in the configuration of each set. Baking Set #1 consisted of three bakers who followed a standard recipe with aliquots of the same ingredients (i.e., same brand and lot numbers) but using different ovens, balances, rolling pins, and water amounts. Baking Set #2 involved five bakers using the same recipe and ingredients, but with more controlled parameters: baking powder and salt were weighed more accurately in the laboratory and an adjustable rolling pin was used to achieve a uniform thickness of the dough before cutting the shapes. Baking Set #3 aimed to introduce more variations in the baking parameters to assess the variability of the formed biscuits. This set included four bakers who were not directly involved in the study to reduce potential bias during preparation. The bakers were provided with the recipe but without the ingredients (except for the 10 g of defatted chia flour) and were asked to prepare the biscuits as they would do in real life, modifying as needed and reporting any changes made to the recipe.

**Fig. 1:**
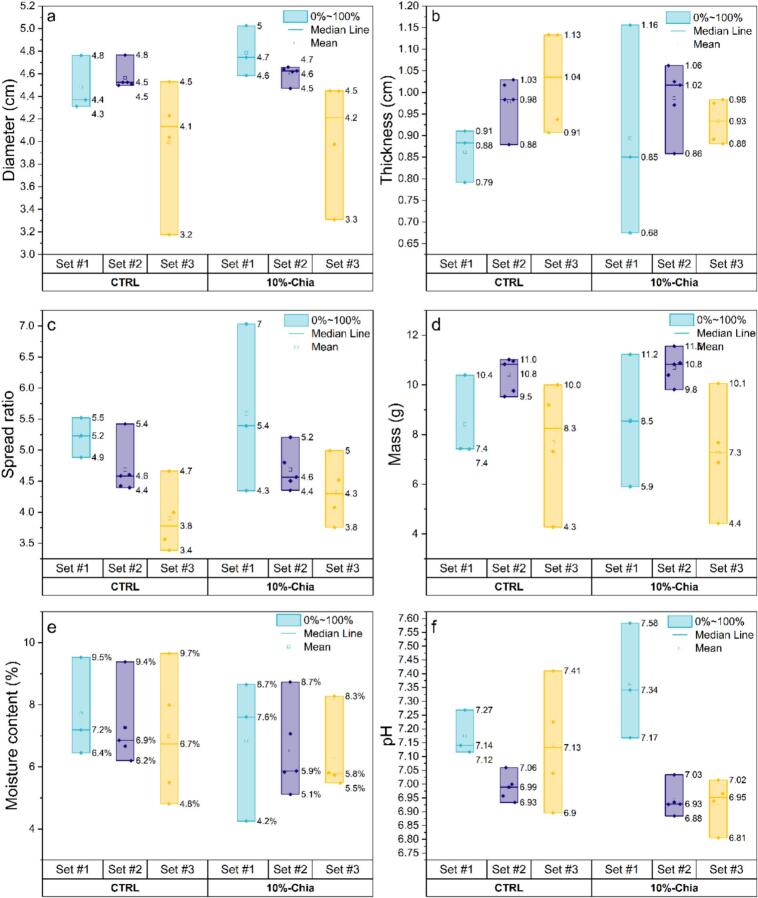
Comparison of the physico-chemical parameters (a) diameter, b) thickness, c) Spread ratio, d) mass, e) moisture content and f) pH) as a function of the type of biscuit (CTRL and 10 %-Chia) and the baking set. For each boxplot all the bakers are included (three for Set #1, five for Set #2 and four for Set #3).

Fig. 1 presents the physical parameters for the CTRL and 10 %-Chia conditions in each baking set, with each box representing the variability between all the bakers. Firstly, it can be observed that the physical parameters of the most controlled set (#2) exhibit the highest level of homogeneity, characterised by small variabilities both between bakers and between the CTRL and 10 %-Chia conditions. The diameters, masses and the pH values showed particularly low variability in Set #2, which is likely because only the baker, the oven, and the amount of water were varied, while other parameters were kept constant. There were fewer differences in thickness and spread ratio, with only two out of ten batches differing from the majority (Baker E – CTRL and Baker B – 10 %-Chia) (Fig. S3b and S3c).

The diameter showed significant variation only in the baking Set #3. These differences were observed between the bakers, who employed varying shape forms, but not between the CTRL and 10 %-Chia groups (Table S7 and Figs. S2, S3 and S4).

In terms of thickness, the highest variation between bakers was observed for the 10 %-Chia in Set #1. The thicknesses in the CTRL group were fairly uniform, with only baker A showing a significant difference from the other two at *p* < 0.05 (Fig. S2b). When comparing the CTRL group with the 10 %-Chia group, only Baker A maintained similar thicknesses with and without chia.

Similarly, the highest variation in spread ratio is observed for the 10 %-Chia in Set #1. The spread ratio values showed that only Baker B – 10 %-Chia and Baker C – 10 %-Chia differed from the other samples (including CTRL) (Fig. S2c). Baker C – 10 %-Chia had a significantly lower spread ratio than the others, which means that the biscuits rose much more considering that the diameter was quite homogeneous. On the other hand, Baker B - 10 %-Chia has a significantly higher spread ratio, confirming from the thickness values that Baker B had rolled this dough much more than the control.

The mass variation was clearly greater in Set #1 and Set #3 (Fig. 1). In Set #3, these differences were primarily observed between the bakers and less between the CTRL and the 10 %-Chia, similar to the diameter data. This can be attributed to the different baking shapes used to form the biscuits (Fig. S4d). In Set #1, the mass measurements provided additional information and showed that the difference between Baker C – CTRL biscuits and Baker C – 10 %-Chia biscuits was small. This suggests that, while the mass and diameter remain relatively constant, the increased thickness of the 10 %-Chia biscuits may indicate that a higher baking powder amount was used. This hypothesis was confirmed by the pH measurements, with values of 7.14 ± 0.05 and 7.58 ± 0.02 for Baker C – CTRL and Baker C – 10 %-Chia, respectively (Fig. S5, Table S7). In the case of Baker B, the lower mass of the chia biscuits compared to the control confirms that the biscuits were thinner, likely due to the preparation with the rolling pin, proving the difficulty in controlling thickness by using a conventional rolling pin without spacers.

Regarding the moisture content, Fig. 1e does not show significant differences in values and data variability as a function of the sets or the type of biscuits (CTRL and 10 %-Chia).

The pH values of the biscuit extracts ranged from 6.8 to 7.6, indicating near-neutral to slightly basic conditions (Fig. 1f). Moreover, significant differences in pH values and variability were observed between sets and biscuit types. This variability could be attributed to the high weight uncertainty associated with small masses in Set #1 and Set #3, specifically for the 1 g of baking powder. The pH of Set #2 CTRL and 10 %-Chia were equivalent (mean, median, min and max).

### 3.2 Acrylamide content

#### 3.2.1 Comparison of AA formation in the different baking sets

**Fig. 2:**
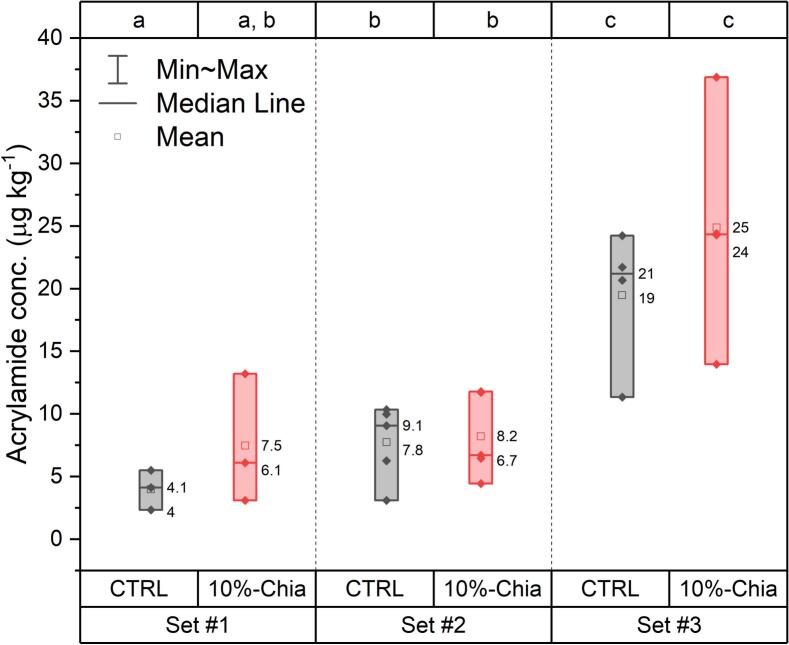
AA variation in control biscuits and biscuits containing 10 %-Chia for the three sets (Set # 1: three bakers, Set #2: five bakers and Set #3: three bakers). On the top axis, the results of independent samples *t*-tests are displayed, which were conducted to compare each group pairwise. Different letters indicate which groups are statistically different at *p* < 0.05 using t-test for independent samples (Details in Table S4).

**Fig. 3:**
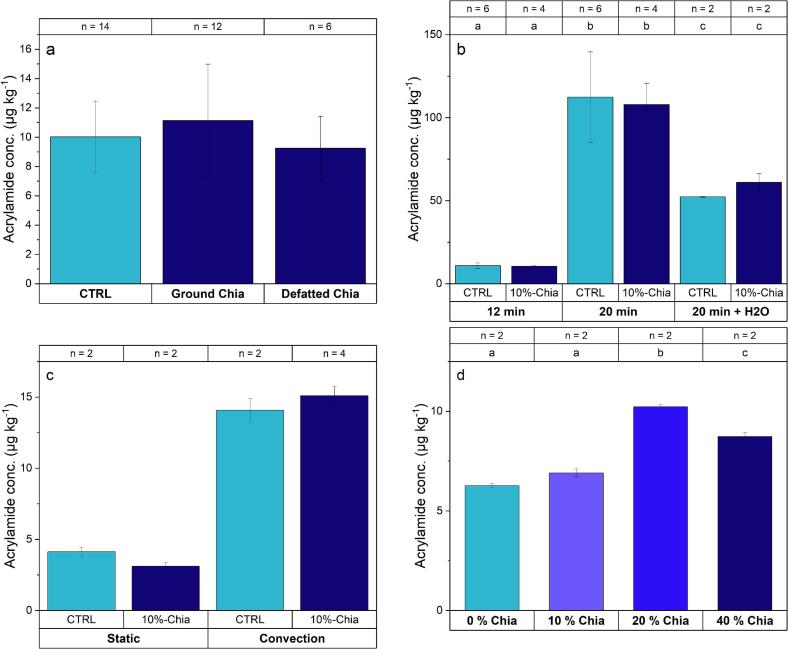
AA content in control biscuits and biscuits containing 10 %-Chia a) Comparison between the type of chia: ground seeds and defatted flour (*T* = 180 °C, baking time = 12 min, convection oven, prepared by different bakers), b) Comparison of baking time (T = 180 °C, defatted chia flour used, average of AA content prepared by two different bakers, convection oven) and baking time of 20 min with a glass of water in the oven (only one baker), c) Comparison of oven mode: static and convection oven-baked biscuits (T = 180 °C, baking time = 12 min, ground chia used, prepared by one baker on the same day), d) Comparison of different percentages of chia incorporation (T = 180 °C, baking time = 12 min, convection oven, defatted chia flour used, prepared by one baker on the same day). n represents the number of replicates of extraction used to calculate the mean + STD. The uncertainty is calculated as one STD. the letters (a, b, c) indicate if the groups are statistically different at *p* < 0.05 using an independent samples *t*-test.

This aim of this section is to assess the impact of adding chia (ground seeds or defatted flour) on the AA content of biscuits, taking into account the high degree of variability in baking conditions. Fig. 2 summarises the AA concentrations for the CTRL and the 10 %-Chia samples in the different baking sets. The AA contents were relatively low, with values below 40 μg kg^−1^, which are significantly lower than the benchmark level of 350 μg kg^−1^ established for biscuits (EU Regulation 2017/2158, 2017). The low AA levels are likely due to the biscuit formulation and cooking conditions. The resulting biscuits were not crispy, had a light colour, and were approximately 1 cm thick. Across all baking sets, the CTRL and 10 %-Chia groups showed no statistically significant differences in AA content, as confirmed by independent samples *t*-tests (see Table S4 for details). It should be noted that defatted chia flour was used in this set, in contrast to the other sets where ground chia was used. However, as can be seen in Fig. 3a, no significant difference was observed between the two types of chia. The concentrations in Set #3 had greater variability and were significantly higher (mean around 19 and 25 μg kg^−1^) than the two other sets, likely due to the fact that the bakers in this set tended to increase the baking time (12–18 min instead of the standard 12 min based on their personal preference (Table S2). This tendency is similar to what Mesías et al. (2020) observed for French fries, where consumers tended to cook their food longer (Mesías et al., 2020). The difference between Set #1 and Set #2 was smaller, with only the CTRL group in Set #1 being significantly lower from the CTRL and 10 %-Chia groups in Set #2. This difference is likely due to the use of convection oven mode in Set #2 instead of static mode in Set #1. In contrast, the 10 %-Chia group in Set #1 did not show a statistically significant difference with the Set #2 groups, probably because the variation in AA concentration was larger.

A more detailed analysis of the data on a baker-by-baker basis (Fig. S6 and Table S5) indicated that significant differences between the CTRL and the 10 %-Chia conditions could occur, but these differences were not systematic, and the 10 %-Chia biscuits did not consistently show higher AA levels. Instead, the results depended on the individual baker and on the biscuit preparation:

- In Set #1, Baker A had a significantly lower AA content in the 10 %-Chia biscuits (3.11 ± 0.05 μg kg^−1^) compared to the CTRL biscuits (4.12 ± 0.06 μg kg^−1^) probably due to their higher thickness (0.85 ± 0.04 cm vs. 0.79 ± 0.03 cm) and the related moisture content (7.60 ± 0.05 % vs. 6.45 ± 0.03 %). In contrast, Baker B and C exhibited a higher AA content in the 10 %-Chia biscuits, with values more than twice as high as those in the CTRL biscuits. As discussed in section 3.1, the differences in processing conditions may have contributed to these variations. Specifically, Baker B's more vigorous dough rolling resulted in thinner and lighter biscuits with a higher surface area ratio compared to the CTRL, leading to an increased AA content (13.2 ± 0.2 μg kg^−1^ vs 5.5 ± 0.2 μg kg^−1^). In contrast, the main difference between Baker C's 10 %-Chia (6.1 ± 0.2 μg kg^−1^) and the CTRL (2.35 ± 0.07 μg kg^−1^) biscuits was presumably the higher amount of baking powder used, influenced by the kitchen balance precision, which increased the pH level (section 3.1). Research has shown that the addition of acids (e.g. citric acid, lactic acid, or tartaric acid) is an effective method of reducing AA in bakery products (Keramat et al., 2011). For example, a study by Hölzle et al. (2025) found that adding citric acid or ascorbic acid to biscuit formulations reduced AA formation by at least a factor of two. On the other hand, under alkaline conditions, the Maillard reaction is promoted by deprotonating the free amino acid, which results in the formation of nucleophilic molecules that can easily react with the carbonyl group, leading to the formation of glycosylamine, an intermediate compound that ultimately triggers the production of AA (Govindaraju et al., 2024). Pearson correlation coefficients were calculated to assess the relationship between AA concentrations and the various biscuit parameters (Table 3). The strongest correlations were found with biscuit mass (*r* = −0.58), spread ratio (*r* = 0.69) and moisture content (*r* = −0.81), which confirm the previous observations. The very strong correlation with the moisture content indicates that lowering the humidity in biscuits leads to increased AA concentrations. The relationship between moisture content and AA formation is well established in the literature, where studies have shown that the presence of water can limit AA formation by keeping the temperature below 100 °C during the baking process (especially in the inner parts of the biscuits) (Açar & Gökmen, 2009; Adimas et al., 2024; Govindaraju et al., 2024). Additionally, a moderate positive correlation was also observed with pH, consistent with previous findings.

**Table 3:** Pearson correlation coefficients of AA with the different biscuit parameters for each set (the entire correlation matrices with correlation coefficient between all the parameters are in Table S6), * significance level at *p* < 0.05.

	Set #1	Set #2	Set #3
*Pearson's correlation coefficient*	AA	AA	AA
Mass biscuit	−0.58	−0.12	−0.62
Diameter	0.12	−0.56	−0.2
Thickness	−0.4	−0.11	−0.75*
Spread ratio	0.69	−0.11	0.51
Weight loss	0.36	−0.14	−0.19
Moisture	−0.81*	−0.81*	−0.31
pH	0.47	−0.53	−0.38
- In Set #2, the baking parameters were subject to higher control, resulting in fewer differences between the CTRL and 10 %-Chia formulations for each baker, with the exception of Baker B. The main factor influencing the AA formation in this set was the moisture content as demonstrated by a strong correlation coefficient of −0.81. This confirms that higher moisture content is associated with reduced AA formation. In contrast to Set #1, the pH level showed a moderate negative correlation with AA concentration (−0.53). Although this correlation is statistically significant (*p* < 0.05), its practical significance should be considered in the context of the limited pH variation in Set #2 (0.2 points compared to 0.5 points in Set #1). Furthermore, the high-moisture samples from Baker E may have skewed the correlation towards low AA and high pH values (Table S7).
- If the two sets of data are put together (Set #1 + Set #2, where the same ingredients were used), it confirms that the moisture content is the only parameter that correlates strongly with the AA concentration (r = −0.8, *p* < 0.001), with all other biscuit parameters having a correlation coefficient below 0.2. Therefore, controlling the moisture content during the baking process could be an effective strategy for reducing AA formation.
- In Set #3, a significant difference between CTRL and 10 %-Chia biscuits was observed for Baker H and Baker I. For Baker H, these differences can be explained by the fact that the mass of the 10 %-Chia biscuits was significantly lower (7.7 ± 1.3 g vs. 9.2 ± 1.0 g) as was the moisture (8.28 ± 0.04 % vs. 9.66 ± 0.09 %). In contrast, for Baker I, the biscuit thicknesses did not show significant differences (0.74 ± 0.10 cm vs. 0.82 ± 0.12 cm for the 10 %-Chia and CTRL biscuits, respectively), although surprisingly the moisture content did not show an inverse correlation (5.48 ± 0.02 % vs. 4.81 ± 0.01 %). Nevertheless, a visual examination of the star-shaped biscuits showed that the chia biscuits were more uneven and had darker and thinner points (see photos in Fig. S5 and Fig. S6). As shown in Table 3, the main parameters influencing AA formation in this batch are the dimensional parameter: spread ratio, mass and thickness, rather than moisture. This is likely due to the fact that the specific surface area is much higher, and as AA is mainly formed at the surface of the biscuit and therefore, the content normalized to the entire volume may be higher. It should be noted that this baking set had higher variability in terms of shape, size, oven mode and baking time contributing to the complexity of the results. For example, in the case of Baker F, the mass of the biscuit was significantly lower, approximately half that of the other biscuits. Moreover, Baker F and Baker G have used a static mode oven, which may have formed less AA than in convection mode for the same baking time, as confirmed in Fig. 3c (Schouten et al., 2022). In addition to that, the ingredients used differed; for instance Baker H used a brown sugar which has been reported to form more AA than other types of sugars (Hölzle et al., 2025)*.* These results may be due to brown sugar containing more reducing sugars (such as glucose and fructose), having AA present originally, or having a higher amount of asparagine (Asikin et al., 2017; Henao Toro et al., 2022; Shyu et al., 2019).

In summary, the findings indicate that incorporating 10 % chia does not lead to an increase in AA formation, even under the diverse conditions typical of real-world baking scenarios (different individuals, various kitchen settings, various ingredients). The results suggest that this is the processing conditions and ingredient composition significantly impact AA formation in biscuits, as described in the literature (Keramat et al., 2011). They also highlight the importance of considering the physical properties of the biscuits and the moisture content, in relation to AA formation. The strong negative correlation between AA content and moisture content suggests that moisture plays a critical role in AA formation, and controlling moisture levels can help minimise AA formation in baked goods.

#### 3.2.2 Effects of other parameters on AA formation with chia incorporation

Fig. 3 compares different parameters that may have an impact on the AA formation. First, Fig. 3a shows that the incorporation of 10 % ground chia or defatted chia has no statistically significant effect on AA concentrations. The uncertainties reflect the variability among several bakers across different kitchens. Fig. 3b and Fig. 3c aim to determine whether other factors known to influence AA formation create a distinction between the CTRL biscuits and those containing 10 % chia. The use of a convection oven to bake the biscuits increased the formation of AA (Fig. 3c). This effect may be due to the fact that in this mode the set temperature is reached faster than in static mode and it has a higher heat transfer coefficient, and a quicker humidity loss (Schouten et al., 2022). However, this increase in AA content did not result in significant differences between the CTRL and 10 %-Chia biscuits. Similarly, in Fig. 3b, prolonging the baking time to 20 min led to a 10-fold increase in AA concentration, yet no significant differences were observed between the CTRL and the 10 %-Chia biscuits. It should be noted that a baker-to-baker comparison showed significant differences in AA content between the CTRL and the 10 %-Chia biscuits. However, the trend varied among the bakers, and the highest content was not always associated with the 10 %-Chia biscuits (Fig. S9). Moreover, the biscuits were browner than those baked 12 min and appeared overbaked. Placing a glass of water in the oven to increase the relative humidity and baking for 20 min resulted in around 45 % reduction in AA concentration, providing a simple method of minimizing AA formation. Visually, the biscuits obtained were slightly lighter in colour than those made without added humidity in the oven (Fig. S10).

According to the literature, studies have reported contrasting results regarding the impact of incorporating chia into biscuits on AA formation. In the study by Mesías et al. (2023), replacing wheat flour with chia flour resulted in higher AA levels; the AA concentration was 113 μg kg^−1^ in CTRL biscuits and ranged from 100 to 236 μg kg^−1^ for biscuits containing 5 to 10 % ground and defatted ground chia seeds (Mesías et al., 2023). It was observed that the AA content had a significant negative correlation with moisture content. Their study concluded that replacing wheat flour with chia seeds decreased the moisture content, which therefore increased the AA formation (Mesías et al., 2023). This phenomenon was not observed in our study, knowing that the amount of water added was left to the baker discretion. Data from Hölzle et al. (2025) also indicate that adding ground chia seeds can increase AA levels from 81.1 μg kg^−1^ in the control to 431 μg kg^−1^ in biscuits containing 10 % of milled chia seeds. However, they demonstrated that pH plays an important role in this trend. Specifically, the addition of acids such as 1 % citric acid or ascorbic acid did not increase the AA content of chia biscuits. For instance, with 1 % citric acid, the AA concentration in biscuits without chia seeds was 46.3 ± 2.0 μg kg^−1^, compared to 40.1 ± 1.7 μg kg^−1^ in biscuits with 10 % milled chia seeds. In contrast, the absence of acid resulted in higher AA concentrations in all biscuits (both with and without chia), with the highest concentration observed in chia biscuits containing 10 % milled seeds (241 ± 11 μg kg^−1^ vs. 153 ± 7 μg kg^−1^). Conversely, Verma et al. (2025) found that replacing wheat flour with combinations of buckwheat, chickpea, and chia flours provided better control over AA formation and increased nutritional value (Verma et al., 2025). A screening study on acrylamide levels in various commercial foodstuffs, including cookies, showed that snacks containing chia did not have higher acrylamide levels than other cereal-based snacks (Szternfeld et al., 2025). Overall, no consistent pattern was observed with increasing percentages of added chia. Given the complexity of AA formation in food matrices and the limited number of published studies, it is difficult to establish a definitive relationship based on chia addition alone. It should be noted that the concentrations in the present study are lower than those in the aforementioned papers, likely due to differences in formulation, biscuit thickness (5 mm vs. 2 to 5 mm), baking time (12 min vs. 20–22 min), and temperature (180 °C vs. 180–190 °C).

Fig. 3d shows that increasing the amount of chia in the biscuits (20 % and 40 %) increased significantly the AA formation probably due to higher amount of asparagine in chia than in refined wheat flour as reported in the literature (102 to 237 mg kg^−1^ in wheat flour compared to 257 to 531 mg kg^−1^ in chia flour) (Hölzle et al., 2025; Mesías et al., 2016; Mesías et al., 2023; Verma et al., 2025). However, no direct correlation was observed between AA concentration and chia content. The lower concentration of AA in 40 % chia biscuits compared to 20 % could be attributed to the higher moisture content (8.07 ± 0.02 % vs. 6.93 ± 0.01 %). Moreover, from a textural perspective, the 10 % chia biscuits did not differ drastically from the CTRL biscuits, whereas the 20 % chia and especially the 40 % chia biscuits were more difficult to break, more “elastic”, and chewy, with a more pronounced taste. Therefore, it is unlikely that these high percentages of chia will be incorporated into biscuits to maintain the consistency of a classic biscuit.

## 4 Conclusion

This study examined the influence of adding 10 % chia on AA formation in homemade biscuits, prioritizing the variability encountered in real-life baking scenarios. In total, three sets of baking experiments were performed at 180 °C and 12 min, comprising 24 batches (including controls). The results demonstrated that differences among bakers, ovens, water amounts, ingredient types, and biscuit shapes did not result in substantial differences between control and chia biscuits. The concentrations remained consistently low (2.4 to 37 μg kg^−1^) well below the European benchmark level of 350 μg kg^−1^. Key factors influencing AA levels included moisture content (ranged from 4.3 to 9.7 %), pH (ranged from 6.8 to 7.6), and physical parameters such as biscuit diameter (ranged from 3.2 to 5 cm), thickness (ranged from 0.68 to 1.16 cm), and mass (ranged from 4.3 to 11.2 g).

Extending the baking time from 12 min to 20 min led to a significant 10-fold increase in AA concentration. Additionally, adding a glass of water in the oven reduced AA formation by 45 %, offering a simple method for minimizing AA levels, constituting a practical mitigation strategy for consumers.

Overall, the study provides some insights that chia may be used as an ingredient in homemade biscuit recipes without significantly increasing the risk of AA dietary intake. Additional parameters such as asparagine content, reducing sugar content and traceability of the flours (origin, breed, storage conditions, etc.) are needed to gain a better mechanistic understanding.

## CRediT authorship contribution statement

**Hind El Hadri:** Writing – original draft, Visualization, Methodology, Investigation, Formal analysis, Data curation, Conceptualization. **Ivana Blažević:** Writing – review & editing, Visualization, Investigation. **Ivana Bianchi:** Writing – review & editing, Investigation, Conceptualization. **Otmar Geiss:** Writing – review & editing, Investigation, Conceptualization. **Josefa Barrero-Moreno:** Writing – review & editing, Supervision, Project administration, Methodology, Investigation, Conceptualization.

## Disclaimer

The information and views set out in this study are those of the authors and do not necessary reflect the official opinion of the European Commission. The European Commission does not guarantee the accuracy of the data included in this study. Neither the European Commission nor any person acting on the European Commission's behalf may be held responsible for the use that may be made of the information contained therein.

## Declaration of competing interest

The authors declare that they have no known competing financial interests or personal relationships that could have appeared to influence the work reported in this paper.

## Data Availability

Data will be made available on request.

## Appendix A Supplementary data

Supplementary material Image 1
