# Supplementary material for: Variations in acrylamide content of homemade biscuits with the addition of chia (ground seeds and flour)
Source: Food Chem X. 2025 Oct 15;31:103172. doi: 10.1016/j.fochx.2025.103172 (PMC12554069; doi:10.1016/j.fochx.2025.103172)

Supplementary Materials

Variations in acrylamide content of homemade biscuits with the addition of chia (ground seeds and flour)

Table S1: Nutritional comparison between the chia seed and the chia flour provided by the seller on the label

Nutritional parameters (per 100 g)	Wheat flour Type 00	Chia seeds	Chia flour
**Fat**	0.8 g	36 g	8 g
**Carbohydrate**	73 g	0.8 g	9 g
**Fibre**	2 g	35 g	44 g
**Protein**	9 g	18 g	30 g
**Salt**	0 g	0.01 g	0 g
**Calcium**	Not indicated	645 mg	Not indicated
**Iron**	Not indicated	5.5 mg	Not indicated
**Phosphorus**	Not indicated	811 mg	Not indicated
**Manganese**	Not indicated	8.6 mg	Not indicated

Table S2: ingredients and baking conditions used by the bakers of Set #3

Baking conditions	Baker F	Baker G	Baker H	Baker I
**Flour**	Type 1 (T65) – less refined	Type 00 – most refined	Type 00 – most refined	Type 00 – most refined
**Sugar**	Fine light cane sugar	White sugar	Brown cane sugar	White sugar
**Oil**	Sunflower	Sunflower	Sunflower	
**Baking powder**	Disodium pyrophosphate (E450), Sodium bicarbonate (E500), corn starch	E450i, E500ii, Sodium, potassium and calcium salts of fatty acids (E470a), corn starch	E450, E500, corn starch, E470a, flavours	E450, E500ii, E470a, corn starch, vanilla flavours
**Salt**	Fine pink salt	Iodized fine sea salt	Not indicated	Iodized fine sea salt
**Water**	Mineral (Sant'Anna)	Tap	Tap	Mineral (San Benedetto)
**Water amount**	CTRL: 31 mL<br>10 %-Chia: 30 mL	CTRL: 35 mL<br>10 %-Chia: 33 mL	CTRL: 39 mL<br>10 %-Chia: 45 mL	CTRL: 37 mL<br>10 %-Chia: 37 mL
**Oven mode**	Static	Static	Convection	Convection
**Baking time**	14-15 min	18 min	CTRL: 13.3 min<br>10 %-Chia: 15 min	12 min
**Supplementary information**	Silicon baking mat			

List of blog websites used to create the recipe

Blog-1. (n.d.). *Easy Eggless Vanilla Cookies*. Retrieved April 8, 2025, from https://mommyshomecooking.com/easy-eggless-vanilla-cookies/

Blog-2. (n.d.). *Biscuits « petits beurre » sans beurre*. Retrieved April 8, 2025, from https://dieteticienne-nutritionniste-lyon-caluire.com/recette-minceur-biscuits-petits-beurre-sans-beurre/

Blog-3. (n.d.). *Petit beurre... sans beurre !* Retrieved April 8, 2025, from https://www.quintesens-bio.com/petit-beurre-sans-beurre/

Blog-4. (n.d.). *Petits beurres maison*. Retrieved April 8, 2025, from https://www.grand-fermage.fr/petits-beurres-maison/

Table S3: LC-MS/MS conditions

LC-MS/MS module	Parameter	Setting
**Separation system**	Column	Hypercarb 50 mm x 2.1 mm, 5 µm
	Guard column	Hypercarb 10 mm x 2.1 mm, 5 µm
**HPLC parameters**	Flow rate	0.3 mL min^{-1}
	Column temperature	25 °C
	Injection volume	5 µL
	Mobile phase	A: 0.1 % formic acid in H2O<br>B: Methanol
	Gradient	0-4 min 100 % A<br>4-6 min 100 to 10 % A<br>6-7 min 10 % A<br>7-9 min 10 to 100 % A<br>9-10 min 100 % A
**MS/MS parameters**	Ionization	ESI +
	Mode	MRM
	Monitored transitions	Quantitative transition:<br>72 > 55 for AA<br>75 > 58 for d3-AA<br>Qualitative transition:<br>72 > 44 for AA
	Gas temperature	230 °C
	Gas flow	5 L min^{-1}
	Nebulizer	40 psi
	Sheath gas temperature	400 °C
	Sheath gas flow	11 L min^{-1}
	Capillary voltage	2000 V
	Nozzle voltage	1000 V

Table S4: t-test for independent samples to compare the AA content in each group pairwise. The first three rows compare the CTRL and 10 % Chia groups in each baking set. The last row regroups the CTRL and Chia from all sets. p-values in bold are the pairs that are significantly different.

Pairwise compared	p-value	t	df	Cohen's d	Levene's test
CTRL-Set#1/Chia-Set#1	0.13	-1.76	5.93	1.02	Unequal variance
CTRL-Set#2/Chia-Set#2	0.735	-0.34	18	0.15	Equal variance
CTRL-Set#3/Chia-Set#3	0.122	-1.64	16	0.78	Equal variance
CTRL-Set#1/CTRL-Set#2	**0.004**	-3.47	13.7	1.79	Unequal variance
CTRL-Set#1/Chia-Set#2	**0.003**	-3.63	13.3	1.87	Unequal variance
CTRL-Set#1/CTRL-Set#3	**<0.001**	-7.41	10.5	3	Unequal variance
CTRL-Set#1/Chia-Set#3	**<0.001**	-5.78	12	3.12	Equal variance
Chia-Set#1/CTRL-Set#2	0.884	-0.15	14	0.08	Equal variance
Chia-Set#1/Chia-Set#2	0.707	-0.38	14	0.2	Equal variance
Chia-Set#1/CTRL-Set#3	**0.001**	-3.97	14	2.05	Equal variance
Chia-Set#1/Chia-Set#3	**0.001**	-4.43	12	2.39	Equal variance
CTRL-Set#2/CTRL-Set#3	**<0.001**	-5.26	18	2.35	Equal variance
CTRL-Set#2/Chia-Set#3	**<0.001**	5.88	16	2.79	Equal variance
Chia-Set#2/CTRL-Set#3	**<0.001**	-4.95	18	2.21	Equal variance
Chia-Set#2/Chia-Set#3	**<0.001**	-5.64	16	2.68	Equal variance
CTRL-All sets / Chia-All sets	0.358	-0.93	48	0.26	Equal variance

Levene's test is used to assess whether two groups have equal variances which is an important information for the statistical t-test, a p-value < 0.05 means unequal variances while a p-value > 0.05 means equal variances[1]

Table S5: Independent samples t-test to compare AA content for CTRL and 10 % Chia conditions for each Baker in each set.

CTRL vs 10 %-Chia in the following groups	Difference	p-value	t	df	Levene's test
Baker A – Set #1	Statistically significant	**0.004**	16.86	1.97	Unequal variance
Baker B – Set #1	Statistically significant	**0.001**	-43.25	1.89	Unequal variance
Baker C – Set #1	Statistically significant	**0.009**	-27.97	1.31	Unequal variance
Baker A – Set #2	Not statistically significant	0.123	4.39	1.1	Unequal variance
Baker B – Set #2	Statistically significant	**0.006**	-49.33	1.22	Unequal variance
Baker C – Set #2	Not statistically significant	0.145	1.02	-4.2	Unequal variance
Baker D – Set #2	Not statistically significant	0.185	1	3.35	Unequal variance
Baker E – Set #2	Not statistically significant	0.294	-1.78	1.19	Unequal variance
Baker A – Set #3	Not statistically significant	0.963	0.05	3	Equal variance
Baker B – Set #3	Not statistically significant	0.464	-0.84	3	Equal variance
Baker C – Set #3	Statistically significant	**0.041**	-14.7	1.02	Unequal variance
Baker D – Set #3	Statistically significant	**0.003**	-22.47	1.85	Unequal variance

[1] DATAtab Team (2025). DATAtab: Online Statistics Calculator. DATAtab e.U. Graz, Austria. URL https://datatab.net

Table S6: Pearson correlation matrices of different biscuit parameters for each set (* significance level $p < 0.05$)

	Mass	Diameter	Thickness	Spread ratio	Weight loss	Moisture	pH	AA
**Set #1**								
**Mass**	1							
**Diameter**	**0.68**	1						
**Thickness**	**0.89***	**0.56**	1					
**Spread ratio**	**-0.78***	-0.16	**-0.89***	1				
**Weight loss**	-0.09	0.33	-0.35	**0.54**	1			
**Moisture**	**0.91***	0.37	**0.76***	**-0.83***	-0.19	1		
**pH**	0.33	**0.61**	**0.62**	-0.28	-0.12	0.01	1	
**AA**	**-0.58**	0.12	-0.4	**0.69**	0.36	**-0.81***	0.47	1
**Set #2**								
**Mass**	1							
**Diameter**	-0.25	1						
**Thickness**	**0.88***	-0.25	1					
**Spread ratio**	**-0.84***	**0.53**	**-0.95***	1				
**Weight loss**	**-0.93***	0.41	**-0.86***	**0.88***	1			
**Moisture**	-0.12	**0.54**	-0.03	0.21	0.27	1		
**pH**	-0.26	0.33	-0.22	0.31	0.35	**0.89**	1	
**AA**	-0.12	**-0.56**	-0.11	-0.11	-0.14	**-0.81***	**-0.53**	1
**Set #3**								
**Mass**	1							
**Diameter**	**0.81***	1						
**Thickness**	**0.59**	0.07	1					
**Spread ratio**	0.09	**0.63**	**-0.72***	1				
**Weight loss**	-0.33	-0.3	-0.46	-0.01	1			
**Moisture**	0.52	0.15	**0.82***	-0.48	**-0.88***	1		
**pH**	0.32	0.09	**0.74***	-0.46	-0.38	**0.71***	1	
**AA**	**-0.62**	-0.2	**-0.75***	**0.51**	-0.19	-0.31	-0.38	1

Table S7: Detailed biscuits parameters for all sets, sample types (CTRL and 10 % Chia) and bakers

Set	Baker	Type	Mass of water (g)	Mass before baking (g)	Mass after baking (g)	Biscuit number	Mass biscuit (g)	Diameter (cm)	Thickness (cm)	Spread ratio	Weight loss (%)	Moisture (%)	pH	AA (µg kg^{-1})
Set #1	A	CTRL	23.5	139	111	16	7.4 ± 0.5	4.4 ± 0.2	0.79 ± 0.03	5.5 ± 0.5	19.6	6.45 ± 0.03	7.12 ± 0.02	4.12 ± 0.06
Set #1	A	10 % Chia	23.5	120	103	12	8.5 ± 1.2	4.6 ± 0.2	0.85 ± 0.04	5.4 ± 0.5	14.3	7.6 ± 0.05	7.168 ± 0.003	3.11 ± 0.05
Set #1	B	CTRL	26	114	97	13	7.4 ± 0.7	4.3 ± 0.2	0.88 ± 0.02	4.9 ± 0.3	15.2	7.19 ± 0.01	7.27 ± 0.02	5.5 ± 0.2
Set #1	B	10 % Chia	30	107	83	14	5.9 ± 1.1	4.7 ± 0.2	0.68 ± 0.03	7 ± 0.6	22.8	4.3 ± 0.5	7.34 ± 0.02	13.2 ± 0.2
Set #1	C	CTRL	30	189	146	14	10.4 ± 0.8	4.8 ± 0.4	0.91 ± 0.02	5.2 ± 0.6	23.0	9.53 ± 0.04	7.14 ± 0.04	2.35 ± 0.07
Set #1	C	10 % Chia	35	190	157	14	11.2 ± 1	5 ± 0.3	1.16 ± 0.02	4.3 ± 0.3	17.3	8.66 ± 0.02	7.58 ± 0.02	6.1 ± 0.2
Set #2	A	CTRL	30	181	153	14	11 ± 0.3	4.5 ± 0.3	1.03 ± 0.04	4.4 ± 0.5	15.2	6.67 ± 0.03	6.989 ± 0.002	9.1 ± 0.2
Set #2	A	10 % Chia	33	181	151	14	10.8 ± 0.3	4.6 ± 0.2	1.03 ± 0.03	4.5 ± 0.3	16.5	7.07 ± 0.01	6.93 ± 0.07	6.7 ± 0.7
Set #2	B	CTRL	31	180	152	14	10.8 ± 0.9	4.5 ± 0.5	0.98 ± 0.05	4.6 ± 0.7	15.8	6.86 ± 0.09	6.957 ± 0.03	6.3 ± 0.2
Set #2	B	10 % Chia	24	181	147	15	9.8 ± 0.9	4.5 ± 0.3	0.86 ± 0.03	5.2 ± 0.6	18.7	5.12 ± 0.05	6.93 ± 0.01	11.72 ± 0.05
Set #2	C	CTRL	30	178	146	15	9.8 ± 0.5	4.5 ± 0.2	0.98 ± 0.03	4.6 ± 0.3	17.7	6.2 ± 0.03	6.934 ± 0.009	10.34 ± 0.05
Set #2	C	10 % Chia	28	173	146	14	10.4 ± 0.7	4.7 ± 0.1	0.97 ± 0.04	4.8 ± 0.3	15.9	5.84 ± 0.02	6.928 ± 0.007	11.8 ± 0.5
Set #2	D	CTRL	29	166	143	13	11 ± 0.6	4.5 ± 0.2	1.02 ± 0.01	4.4 ± 0.2	13.7	7.3 ± 0.2	7.00 ± 0.02	9.99 ± 0.03
Set #2	D	10 % Chia	25	159	139	12	11.6 ± 0.6	4.6 ± 0.1	1.06 ± 0.02	4.4 ± 0.1	12.8	5.87 ± 0.03	6.885 ± 0.008	6.5 ± 1.5
Set #2	E	CTRL	41	170	133	14	9.5 ± 0.6	4.8 ± 0.2	0.88 ± 0.05	5.4 ± 0.6	21.5	9.38 ± 0.02	7.06 ± 0.02	3.1 ± 1.0
Set #2	E	10 % Chia	26	166	141	13	10.9 ± 0.8	4.6 ± 0.2	1.02 ± 0.05	4.6 ± 0.4	14.8	8.73 ± 0.06	7.03 ± 0.06	4.5 ± 0.3
Set #3	F	CTRL	31	191	164	36	4.3 ± 0.6	3.2 ± 0.2	0.94 ± 0.03	3.4 ± 0.3	14.1	5.5 ± 0.2	7.040 ± 0.003	24 ± 4
Set #3	F	10 % Chia	30	181	158	33	4.4 ± 0.7	3.31 ± 0.08	0.88 ± 0.04	3.8 ± 0.3	12.7	5.73 ± 0.07	6.967± 0.04	24.4 ± 0.1
Set #3	G	CTRL	35	180	160	14	10.0 ± 2.5	4.5 ± 0.3	1.13 ± 0.02	4 ± 0.3	11.1	7.99 ± 0.03	7.41 ± 0.04	11 ± 4
Set #3	G	10 % Chia	33	181	156	14	10.1 ± 1.7	4.4 ± 0.2	0.98 ± 0.04	4.5 ± 0.4	13.8	5.82 ± 0.2	6.805 ± 0.007	14.0 ± 0.2
Set #3	H	CTRL	30	178	163	17	9.2 ± 1.0	4 ± 0.3	1.13 ± 0.02	3.6 ± 0.3	8.4	9.66 ± 0.09	7.23 ± 0.05	20.7 ± 0.03
Set #3	H	10 % Chia	45	173	161	20	7.7 ± 1.3	4 ± 0.1	0.98 ± 0.12	4.1 ± 0.6	6.9	8.28 ± 0.04	7.02 ± 0.09	24.3 ± 0.3
Set #3	I	CTRL	37	190	-	17	7.3 ± 0.6	4.23 ± 0.09	0.8 ± 0.1	5.2 ± 0.9	-	4.81 ± 0.01	6.896 ± 0.002	21.7 ± 0.6
Set #3	I	10 % Chia	37	190	-	17	6.6 ± 0.9	4.5 ± 0.3	0.7 ± 0.1	6.0 ± 1.2	-	5.48 ± 0.02	6.939 ± 0.003	36.9 ± 0.8

Fig S1. extraction method using SPE from EN 16618:2015 standard method[2]

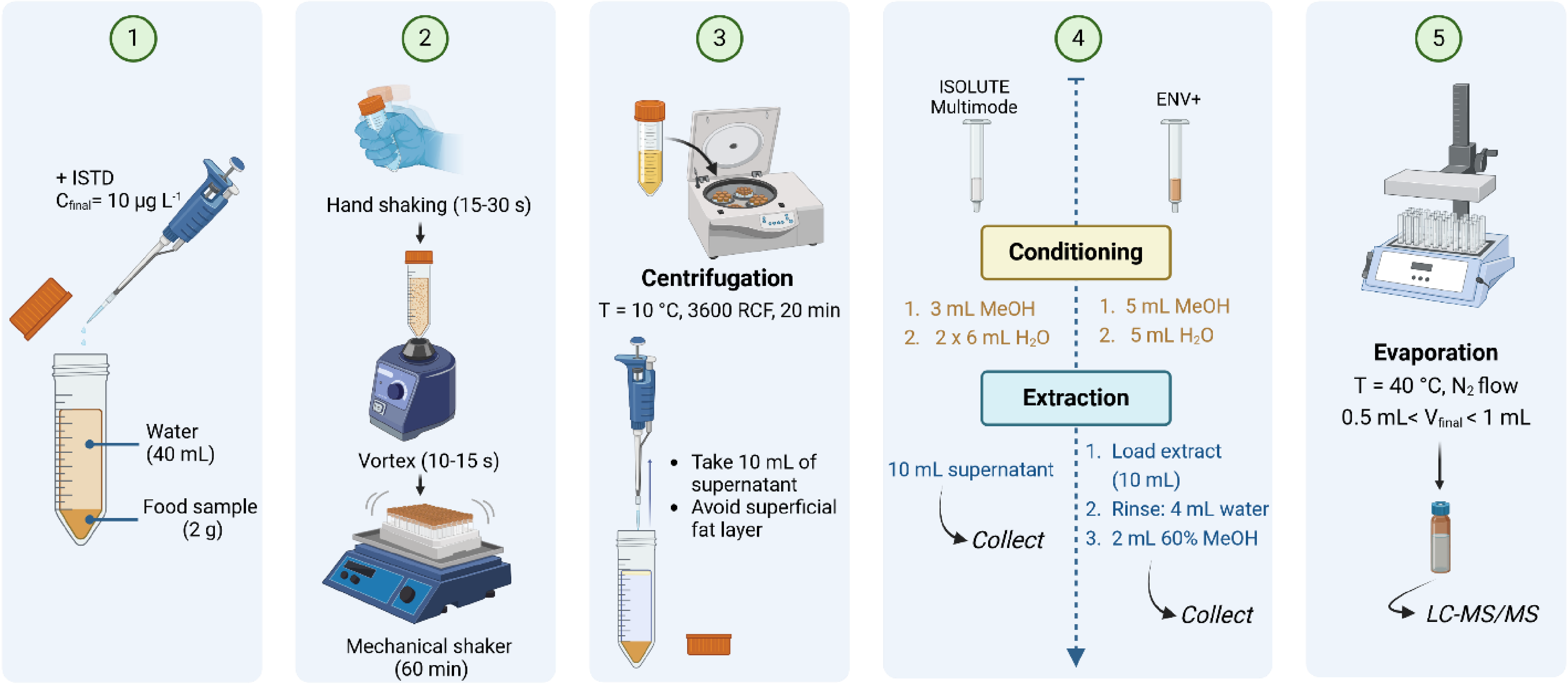

---
[2] Created in BioRender. https://BioRender.com

Fig S2. Physical properties of the biscuits prepared by the three bakers (A, B and C) in Set #1 a) diameter, b) thickness, c) spread ratio and d) mass of one biscuit. The letters shows if the data are significantly different at p<0.05 (Bonferroni test) – At the bottom, photos of the baked biscuits (CTRL at the top, 10 %-Chia at the bottom) in Set #1 for baker A, B and C from the left to the right

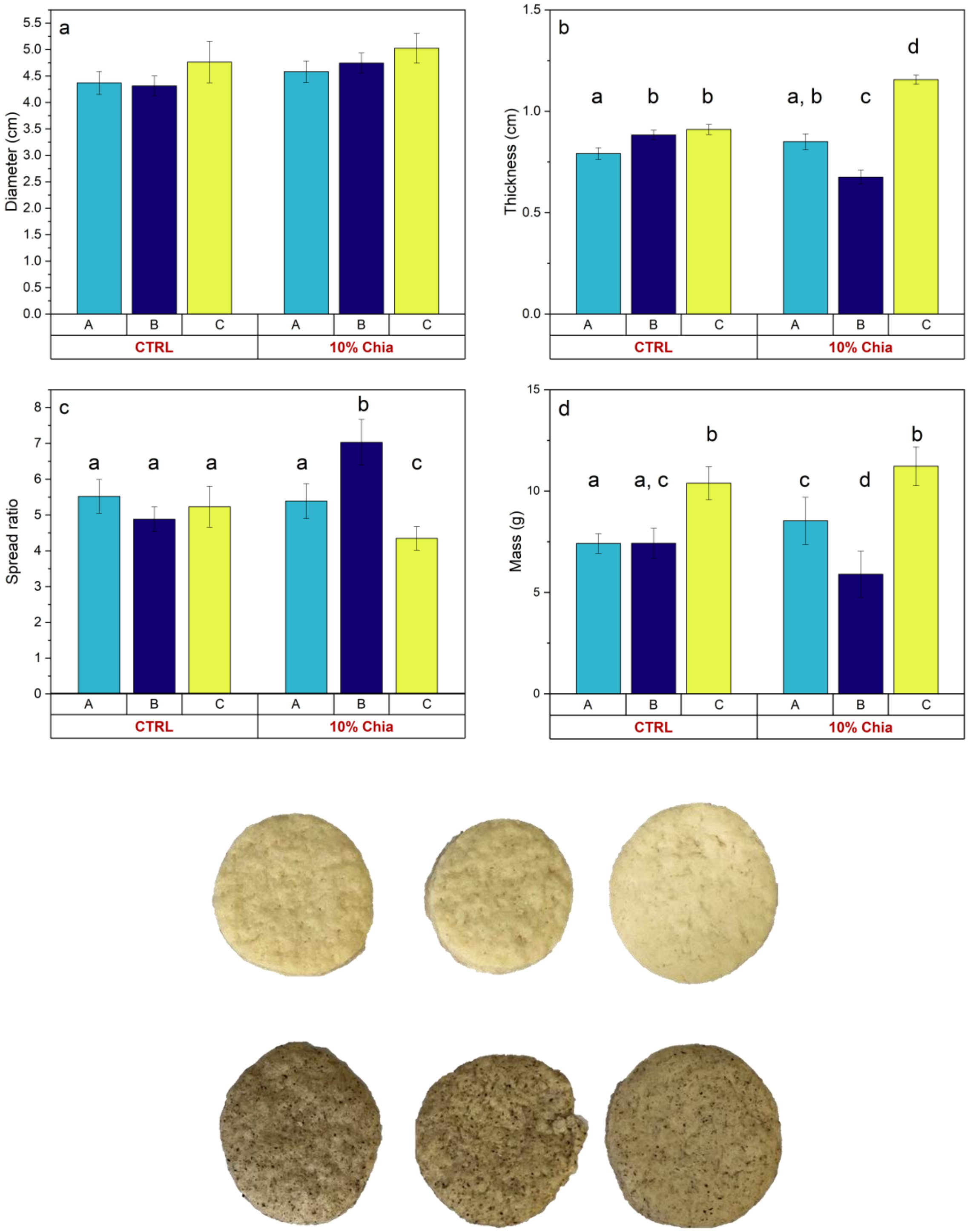

Fig S3. physical properties of the biscuits prepared by the five bakers (A, B, C, D and E) in Set #2 a) diameter, b) thickness, c) spread ratio and d) mass of one biscuit. The letters shows if the data are significantly different at p<0.05 (Bonferroni test) – At the bottom, photos of the baked biscuits (CTRL at the top, 10 %-Chia at the bottom) in Set #2 for baker A, B, C, D and E from the left to the right

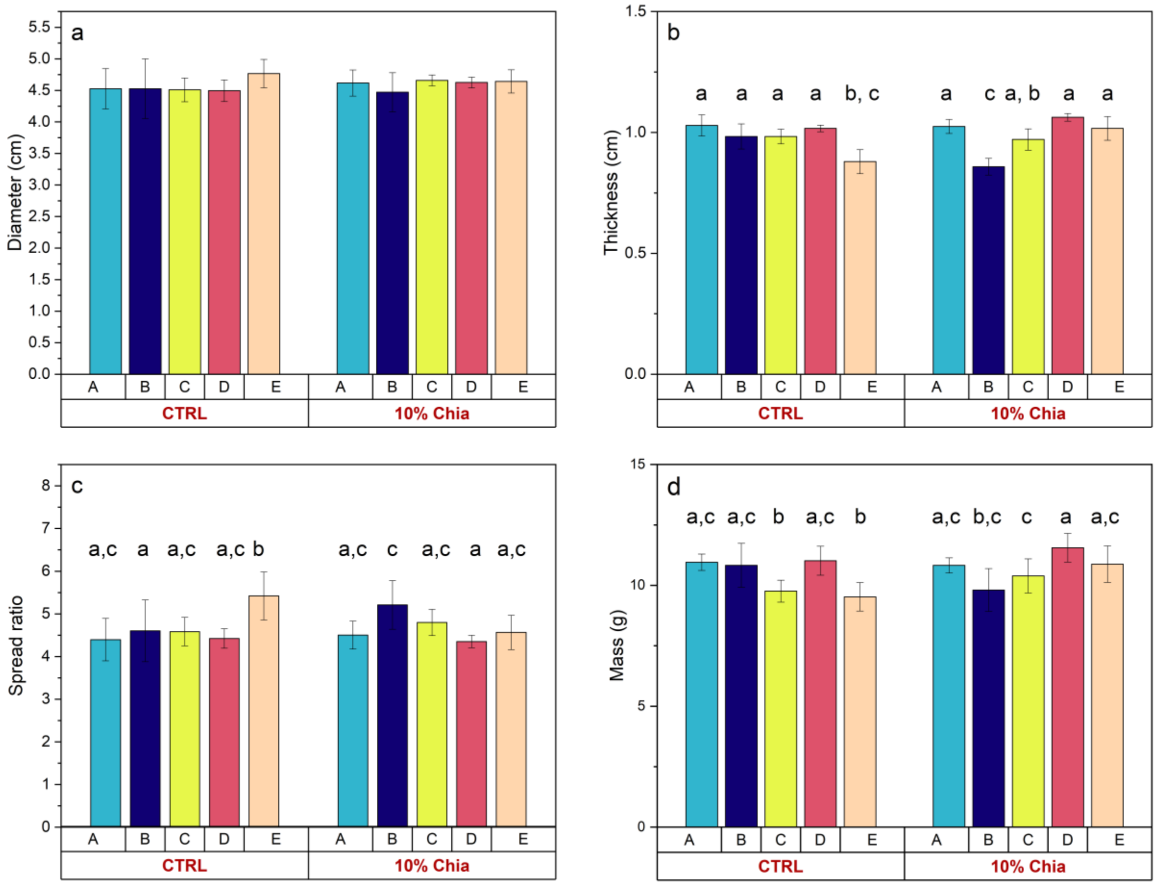

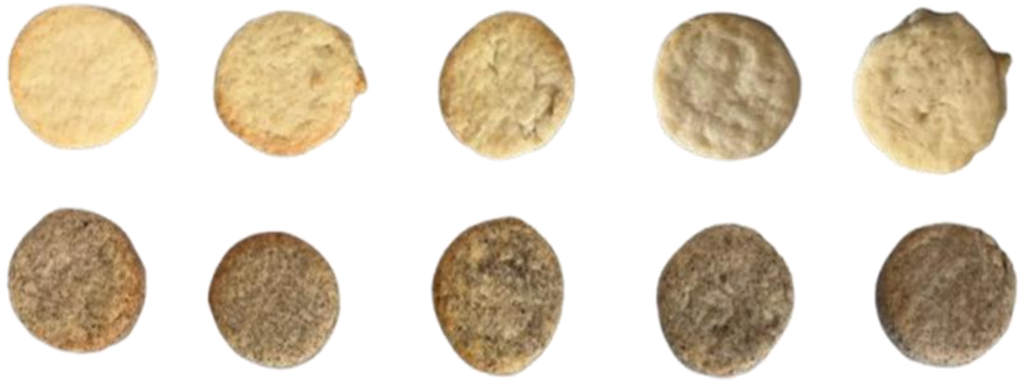

Fig S4. physical properties of the biscuits prepared by the four bakers (F, G, H and I) in Set #3 a) diameter, b) thickness, c) spread ratio and d) mass of one biscuit

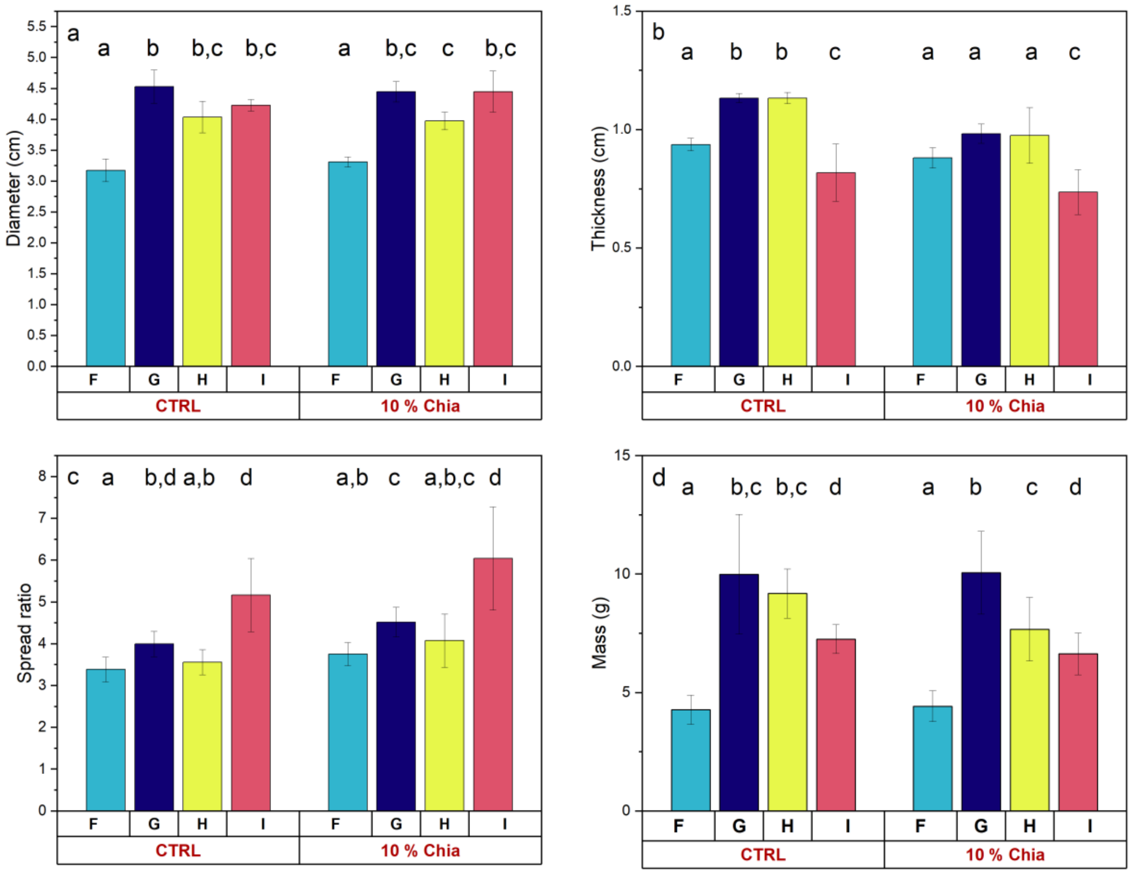

Fig S5. Photos of the baked biscuits (CTRL at the top, 10 %-Chia at the bottom) in Set #3 for baker F, G, H and I from the left to the right (top), zoom on baker I (bottom)

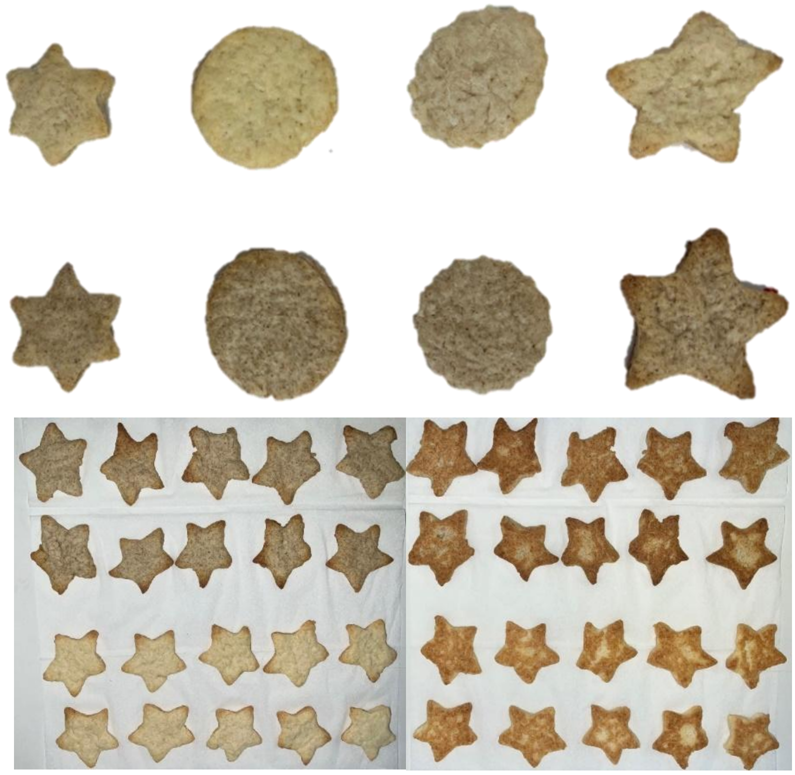

Fig S6. Thicknesses of the star points of Baker I biscuits (lognormal fit)

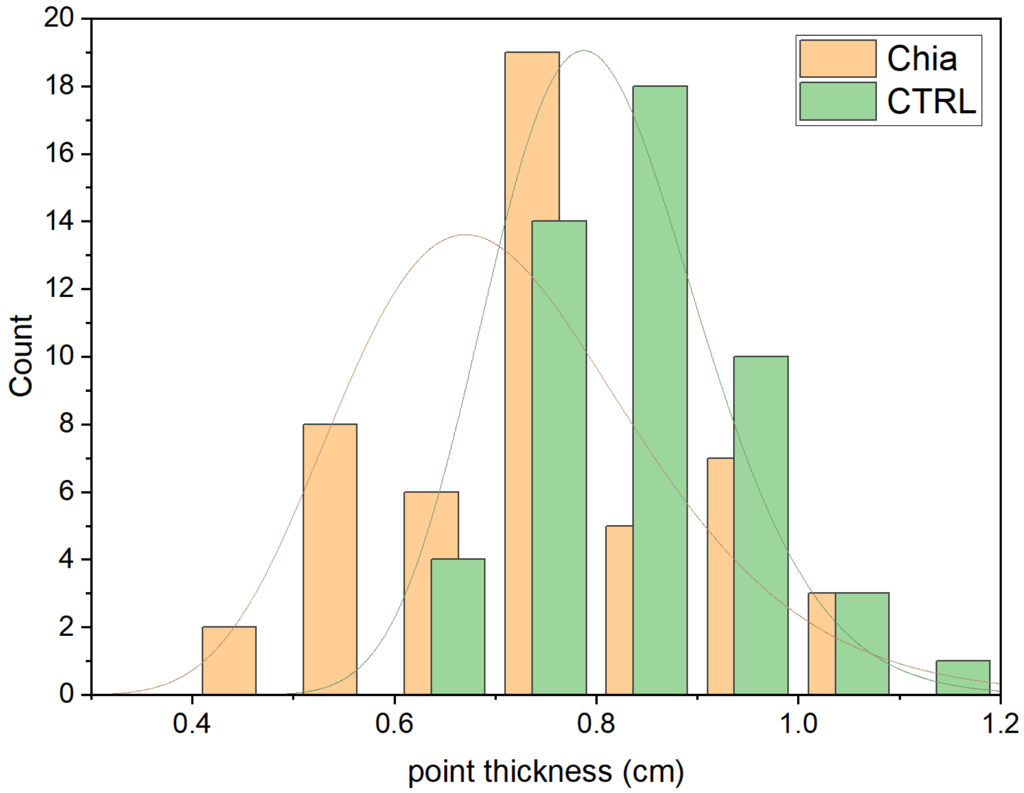

Fig S7. pH of the biscuits of Set #1

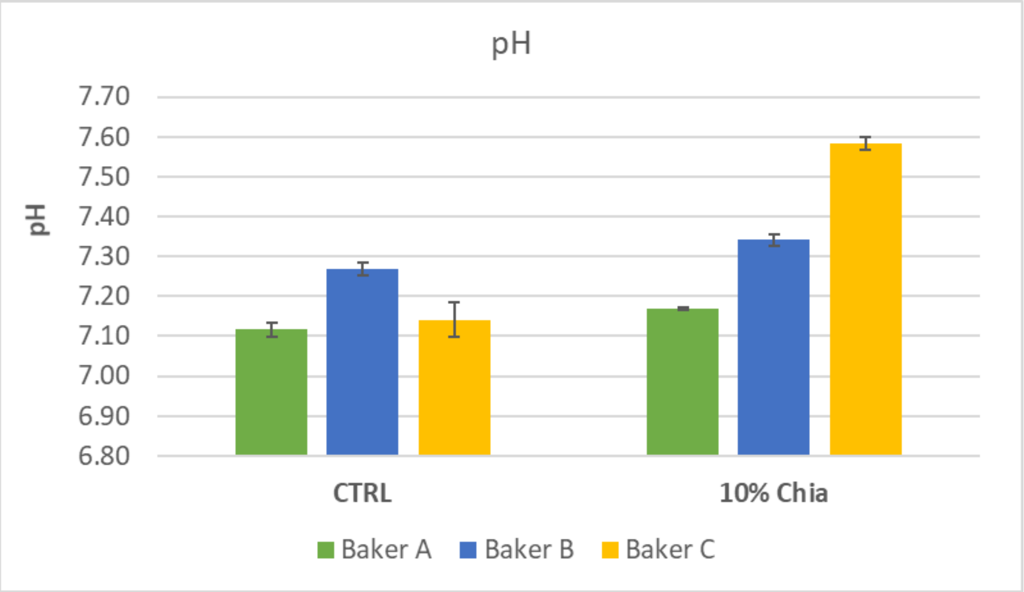

Fig S8. Acrylamide content in control biscuits and biscuits containing 10 % chia for each baker for a) Set #1, b) Set #2 and c) Set #3

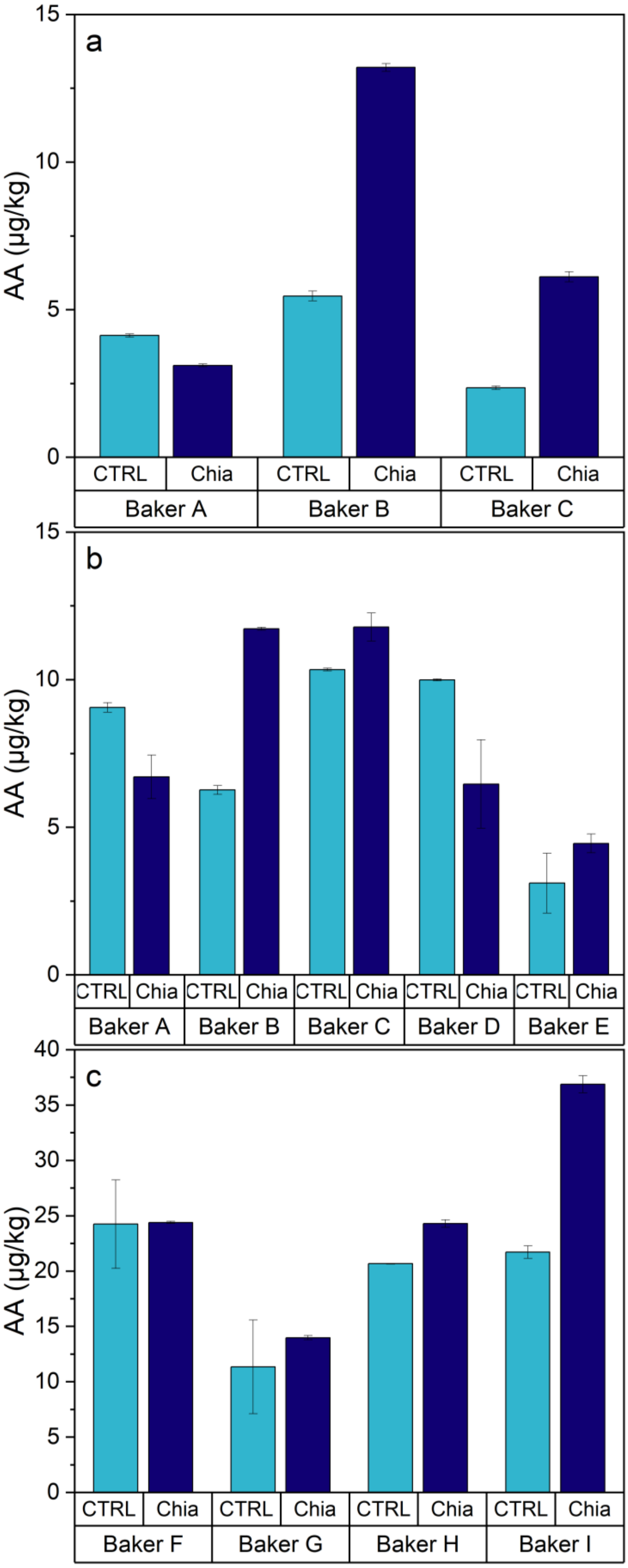

Fig S9. Acrylamide content in control biscuits and biscuits containing 10 % chia for each baker (A and B) for 12 min and 20 min baking time

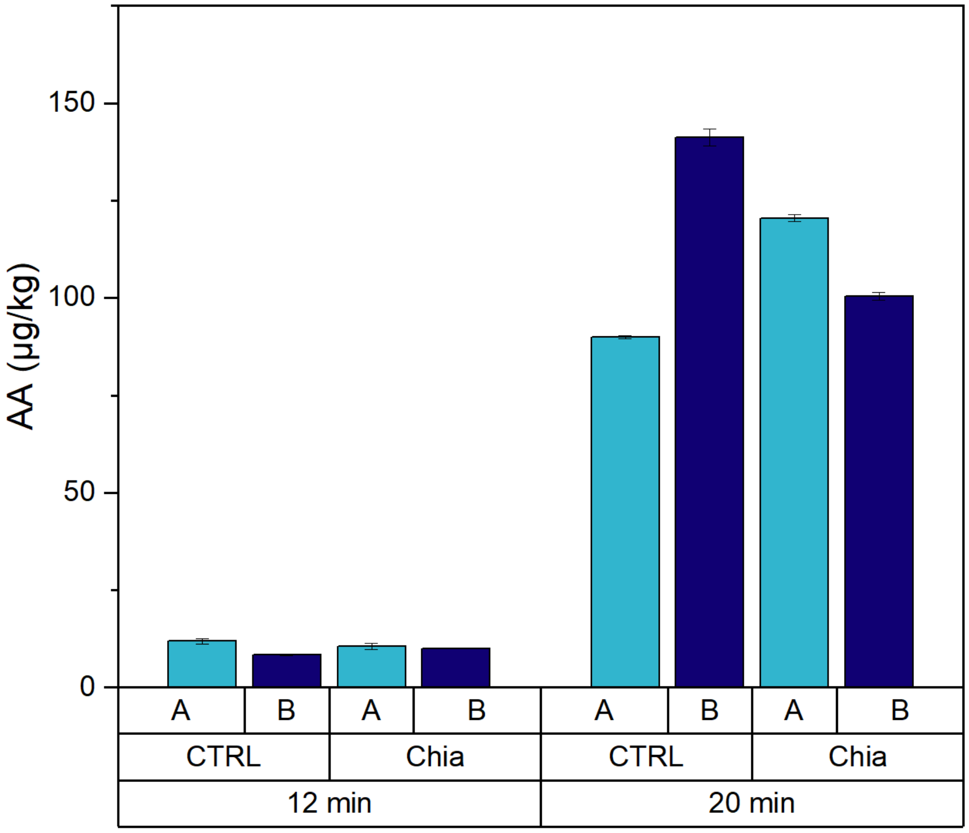

Fig S10. Photos of the biscuits (CTRL at the top, 10 %-Chia at the bottom) for baker A, baked at 180 °C for 20 min, without (left) and with (right) a glass of water in the oven

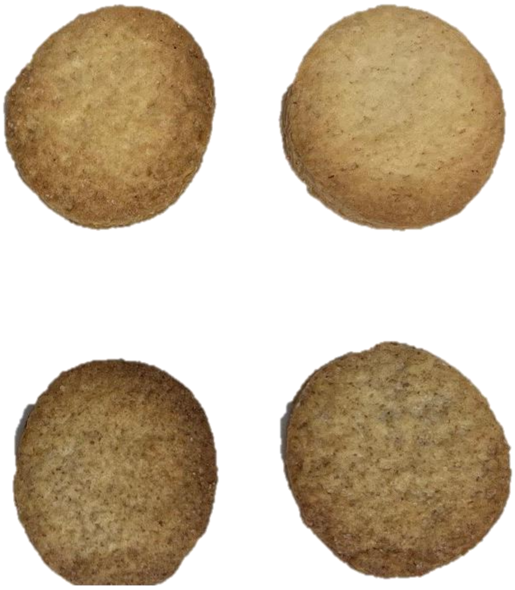

Supplement: Supplementary file 1 — Supplementary material [file mmc1.pdf]
